# The neural basis of hot and cold cognition in depressed patients, unaffected relatives, and low-risk healthy controls: An fMRI investigation

**DOI:** 10.1016/j.jad.2020.05.022

**Published:** 2020-09-01

**Authors:** Nord CL, Halahakoon DC, Lally N, Limbachya T, Pilling S, Roiser JP

**Affiliations:** a Institute of Cognitive Neuroscience, University College London, London, UK; b MRC Cognition and Brain Sciences Unit, University of Cambridge, Cambridge, UK; c Department of Psychiatry, University of Oxford, Oxford, UK; d Warwick Medical School, University of Warwick, Coventry, UK; e Experimental Therapeutics & Pathophysiology Branch, Intramural Research Program, National Institute of Mental Health, National Institutes of Health, Bethesda, MD, 20892, USA; f Camden and Islington NHS Foundation Trust, London, UK; g Department of Clinical, Educational, and Health Psychology, University College London, London, UK

**Keywords:** Depression, DLPFC, Amygdala, Working memory, Emotion processing

## Abstract

**Background:**

Modern cognitive neuropsychological models of depression posit that negatively biased emotional (“hot”) processing confers risk for depression, while preserved executive function (“cold”) cognition promotes resilience.

**Methods:**

We compared neural responses during hot and cold cognitive tasks in 99 individuals: those at familial risk for depression (*N* = 30 unaffected first-degree relatives of depressed individuals) and those currently experiencing a major depressive episode (*N* = 39 unmedicated depressed patients) with low-risk healthy controls (*N* = 30). Primary analyses assessed neural activation on two functional magnetic resonance imaging tasks previously associated with depression: dorsolateral prefrontal cortex (DLPFC) responsivity during the n-back working memory task; and amygdala and subgenual anterior cingulate cortex (sgACC) responsivity during incidental emotional face processing.

**Results:**

Depressed patients exhibited significantly attenuated working memory-related DLPFC activation, compared to low-risk controls and unaffected relatives; unaffected relatives did not differ from low-risk controls. We did not observe a complementary pattern during emotion processing. However, we found preliminary support that greater DLPFC activation was associated with lower amygdala response during emotion processing.

**Limitations:**

These findings require confirmation in a longitudinal study to observe each individual's risk of developing depression; without this, we cannot identify the true risk level of the first-degree relative or low-risk control group.

**Conclusions:**

These findings have implications for understanding the neural mechanisms of risk and resilience in depression: they are consistent with the suggestion that preserved executive function might confer resilience to developing depression in first-degree relatives of depressed patients.

## Highlights

• Depressed patients show decreased prefrontal cortex activation compared to controls.
• First-degree relatives without depression show intact prefrontal cortex activation.
• The prefrontal cortex may be involved in resilience to depression.
• This could also provide preliminary support for targeting this region in treatment.

## 1 Introduction

Family history plays an important role in the development and maintenance of major depressive disorder (MDD): first-degree relatives of patients with MDD have a two-to-fourfold increased risk of developing MDD (Weissman et al., 1993), and MDD tends to onset earlier, and recur more frequently in patients with a family history (Gotlib et al., 2014; Hollon et al., 2006). The cognitive neuropsychological model of depression proposes a causal role for negative affective biases (“hot” emotion processing) in the aetiology of depression (Harmer et al., 2009; Robinson and Sahakian, 2008; Roiser et al., 2012; Roiser and Sahakian, 2013), with preserved executive function mechanisms (“cold” cognition) thought to promote resilience to depression (Roiser et al., 2012) (here, we define resilience to depression as a protective factor promoting a current non-depressed state). It proposes that antidepressant medications act directly on bottom-up affective biases via alterations in monoamine neurotransmission, whilst psychological interventions such as cognitive behavioural therapy (Beck, 1979) have top-down effects on negative schemata instantiated by affected biases (Roiser et al., 2012). Testing this hypothesis would ideally involve measuring both “hot” and “cold” processes in the same individuals.

### 1.2 ‘Hot’ and ‘cold’ neural mechanisms in mdd

There is an extensive literature describing differences in the neural basis of “hot” and “cold” cognition in patients with MDD (Disner et al., 2011; Graham et al., 2013; Hamilton et al., 2012; Hamilton and Gotlib, 2008; MacNamara et al., 2017; Schulze et al., 2019; Wang et al., 2015). This has been measured primarily in three ways: using “hot” emotion processing tasks; using “cold” executive function tasks; and using tasks that address the relationship between prefrontal and limbic regions in depression within the same task (note that even when measured independently, it is difficult to fully separate ‘hot’ and ‘cold’ tasks, since even in many cold cognitive tasks, providing feedback on the task might elicit group differences that are driven by the ‘hot’ processing of feedback rather than the ‘cold’ cognitions themselves). The direction of these effects in MDD is highly inconsistent between studies: a recent meta-analysis reported that about half of studies found greater neural activation in patients with MDD compared to controls (cognition: 17 out of 34 studies; emotion: 33/75), while half reported lower activation (cognition: 17/34; emotion: 32/75) (Müller et al., 2017), which the authors suggested could be due to either a lack of spatial convergence of findings between studies or differences in experimental design and procedure. This lack of convergence in study design and inconsistencies in the directionality of findings limits our ability to draw inferences between reports of abnormalities in specific regions associated with “hot” or “cold” cognitive processing in depression.

Studies investigating differences in the neural basis of emotion processing in patients with MDD often report group differences in two brain structures: the amygdala (which has been reported to show both blunted (Schulze et al., 2019) and enhanced (Victor et al., 2010) responses to negative affective stimuli in depression); and the subgenual anterior cingulate cortex (sgACC) (where diminished deactivation to fearful faces has been reported in both depressed patients (Grimm et al., 2009) and participants at a genetic risk of developing depression (O'Nions et al., 2011)). Studies examining differences in the neural mechanisms of “cold” cognition often report differences in prefrontal regions, particularly dorsolateral prefrontal cortex (DLPFC) during working memory; some studies report DLPFC hyperactivation in MDD (Gärtner et al., 2018; Wang et al., 2015), typically interpreted as ‘inefficiency’, while others report hypoactivation (Baxter et al., 1989; Bench et al., 1993; Korgaonkar et al., 2013; Pu et al., 2011; Siegle et al., 2007).

Measuring “hot” and “cold” cognition during the same task has also proven a fruitful approach to delineating the neural mechanisms underlying these processes in healthy and depressed individuals. Specifically, there appear to be separable neural effects of current or historical depression on working memory with or without valenced emotional stimuli (Berman et al., 2011; Bertocci et al., 2012; Kerestes et al., 2012); for a recent meta-analysis summarising the effect of affective stimuli on working memory in individuals with and without mental health problems, see (Schweizer et al., 2018). In one study assessing these mechanisms with separate tasks, the same group of patients with MDD showed greater amygdala reactivity during an emotional task, and lower DLPFC activation during a separate working memory task (Siegle et al., 2007).

Normalizing DLPFC activation may be a common mechanism across pharmacological (Brody et al., 2001; Fales et al., 2009), psychological (Brody et al., 2001; Goldapple et al., 2004), and somatic treatments for depression (Perrin et al., 2012). Non-invasive brain stimulation interventions for depression almost all target the DLPFC (Blumberger et al., 2018; George et al., 2000; Loo et al., 2012; Nord et al., 2019; Nord and Roiser, 2015), though with mixed results. However, some studies suggest that individual differences in DLPFC activation predict treatment response to interventions directly targeting this region (Nord et al., 2019; Weiduschat and Dubin, 2013).

### 1.3 Neural mechanisms of executive and emotional processing in ‘at-risk’ individuals

Most previous functional magnetic resonance imaging (fMRI) studies in populations at risk of depression have measured either “hot” or “cold” cognitive mechanisms, but seldom both “hot” and “cold” cognition in the same individuals (for example, emotion processing (Chan et al., 2009; Mannie et al., 2011; Monk et al., 2008), or working memory (Watters et al., 2019), in at-risk populations). An important exception which measured interactions between these processes in an at-risk population (within the same task) (Monk et al., 2008), identified disruptions in the neural mechanisms associated with hot cognitive processing when attention (a cold cognitive process) was unconstrained, but no differences in hot cognition when attention was constrained. Instead, when attention was constrained the authors found greater prefrontal activation in the high-risk group, illustrating a potential role for both cognitive systems in maintaining a euthymic state in at-risk populations.

The most comprehensive study to date on the neural basis of executive function in individuals at high familial risk of depression reported lower DLPFC activation during working memory updating, suggesting that vulnerability to depression may be associated with disruption to the neural circuits underlying executive function (Watters et al., 2019). This runs contrary to a previous report that found *greater* activation during working memory in participants at familial risk of depression compared to low-risk controls (Mannie et al., 2010). Of note, both studies reported lower mood in the sample with familial risk of depression compared to low-risk controls (although inclusion of current symptoms as a covariate did not alter the results in the study by Watters and colleagues).

### 1.4 Directly testing the predictions of the cognitive neuropsychological model of depression

The cognitive neuropsychological model of depression posits that top-down ‘cold’ prefrontal mechanisms (subserving executive function) may mediate resilience to depression by dampening down bottom-up ‘hot’ limbic mechanisms (subserving emotional biases), which contribute to risk (Roiser et al., 2012). We designed a study building on previous approaches to directly test the predictions of the cognitive neuropsychological model of depression: that intact executive function mechanisms in the DLPFC might counteract the risk conferred by biases in emotion processing (i.e., greater amygdala activation and lower sgACC activation during negative emotion processing) (Roiser et al., 2012). Therefore, we used two cognitive tasks to separately test the hypotheses that: 1) neural emotion processing abnormalities, frequently observed in depression, would also be evident in unaffected relatives; but 2) neural executive function abnormalities would only be present in currently-depressed patients (and would show preserved function in unaffected relatives). We also used an executive function task without feedback to better dissociate ‘cold’ from ‘hot’ cognitive processing.

### 1.5 Hypotheses

We tested the following specific experimental hypotheses: (1) that patients with MDD and first-degree relatives would show greater amygdala activation and lower sgACC deactivation during fearful face processing compared to low-risk controls; and (2) that patients with MDD would show lower DLPFC activation during working memory processing compared to both first-degree relatives and low-risk controls. We also used correlational analyses to test a secondary prediction, across the full sample of participants: that prefrontal mechanisms (DLPFC activation) may dampen down bottom-up negative emotional biases (i.e., responses to emotional stimuli in the sgACC and amygdala).

## 2 Methods and materials

### 2.1 Participants

Ninety-nine participants (46 males) were recruited through a subject database (30 low-risk controls; 30 first-degree relatives, not related to the sample of MDD patients) and Camden and Islington NHS Foundation Trust (39 unmedicated depressed patients; we recruited an unmedicated sample to avoid effects of antidepressant medication on the blood-oxygen-level-dependant (BOLD) signal) (Wagner et al., 2010). All participants were fluent in English (assessed as having been educated in English; this included non-native speakers educated at university-level in English). During recruitment, we ensured groups did not differ significantly from one another in age or sex, but participants were not matched on an individual level.

All participants were screened for current or past psychiatric disorders using the Mini International Neuropsychiatric Interview (MINI), version 5.0.0 (Sheehan et al., 1998). Exclusion criteria for the low-risk control group and the first-degree relative sample included any Axis I psychiatric disorder other than specific phobia, including substance abuse or dependence. Illegal substance use was prohibited in the six weeks preceding the MRI scan, and standard MRI safety restrictions applied. The low-risk control and first-degree relative groups were administered the Family Interview for Genetic Studies (FIGS), a commonly-used and validated family history interview measure from the National Institute of Mental Health Genetics Initiative (Maxwell, 1992; Phelps et al., 2009; Somanath et al., 2002); we employed the FIGS to screen for history of depression (unipolar and bipolar) in first-degree relatives.

All depressed patients met DSM-IV criteria for a current major depressive episode. Exclusion criteria for the depressed patients were: any history of mania (including hypomanic episodes), substance abuse or dependence (save for a remote history of abuse/dependence restricted to a prior major depressive episode), and use of any psychotropic medication in the previous six weeks. Family history of depression was assessed as part of the clinical interview in depressed patients.

Participants were compensated £10/hour. The study was approved by the London Queen Square NHS Research Ethics Committee (ID: 13/LO/1028).

### 2.2 Clinical and cognitive measures

We collected the following measures: mood, using the Beck Depression Inventory (BDI) and the Hamilton Rating Scale for Depression (HAM-D); anxiety, using the Beck Anxiety Inventory (BAI); and anhedonia, using the Snaith-Hamilton Pleasure Scale (SHAPS), reverse-scored to measure anhedonia. In participants who were native English speakers (*N* = 73), we also measured the Full Scale Intelligence Quotient (FSIQ), calculated using converted scores from the Wechsler Test of Adult Reading (Wechsler, 2001). In depressed patients we recorded age of onset, number of depressed episodes, treatment history, and history of hospitalizations and suicide attempts.

### 2.3 Experimental procedure

Participants attended the laboratory on two days. The first day involved screening for psychiatric conditions and MRI contraindications, and training for the n-back task. To meet criteria for the study, all participants had to pass the training (successfully detect one-back, two-back, and three-back matches on a short task version). On the second day, participants completed the MRI scan. Exclusion criteria were applied to the training session, not the fMRI session. See Supplemental Table 1 for task summary.

#### 2.3.1 n-back working memory task (Lally et al., 2013)

The n-back consisted of a continuous sequence of letters, centrally presented for 1000 ms, interleaved with 500 ms fixation crosses. There were 27 blocks, 18 with 12 1-second letters (900 ms fixation cross displayed between trials) for the 3-back and 1-back, and 9 fixation cross rest blocks. The task was coded in MATLAB (release 2015a for Windows, Mathworks, Natick, MA, USA) using the Cogent Toolbox (http://www.vislab.ucl.ac.uk/cogent_2000.php).

We calculated accuracy (*d'*), defined as:

*d'* = *Z*(hit rate) – Z(false alarm rate) where Z is the inverse of the cumulative Gaussian distribution. Our contrast of interest (3-back>1-back) was selected to maximize recruitment of the working memory network, which in this task been shown to increase with increasing cognitive demand across healthy and depressed patients (Harvey et al., 2005). We tested whether behavioural performance might be driving any group effects by calculating an equivalent behavioural score to the 3-back>1-back fMRI contrast, termed ‘*d'* difference score’ and calculated as (3-back *d'*) – (1-back *d'*); note that in this score, to estimate the inverse of the cumulative Gaussian distribution, we subtracted 0.0001 from each participant's hit rate and added 0.0001 to each participant's false alarm rate, since many participants had hits rates of 1 and/or false alarm rates of 0 in the 1-back condition.

#### 2.3.2 Incidental emotion processing task

Each participant was presented with randomly-ordered male and female faces (in an equal proportion), and were instructed to classify the gender of each face using their index and middle fingers. There were twelve 16 second blocks, four per emotion (happy/fearful/neutral), with eight 2 second stimuli per block and a 16 second central fixation cross between blocks. All face stimuli were sourced from the NimStim Face Stimulus Set (http://www.macbrain.org/resources.htm) (Tottenham et al., 2009). Contrasts of interest were fearful>neutral and happy>neutral faces.

### 2.4 MRI acquisition and analysis

We acquired gradient-echo T2*-weighted images using a Siemens Avanto 1.5 Tesla MRI scanner (32-channel head coil), with 36 slices per volume. For the emotion processing task, slice thickness was 2 mm; slice thickness was 2.5 mm in the n-back task to allow fuller brain coverage including the dorsal prefrontal cortex (see Supplemental Figure 1A and 1B for emotion processing and n-back coverage, respectively). All other parameters were the same across tasks: echo time was 50 ms, repetition time per slice was 87msec, and in-plane resolution was 2 × 2 mm (whole-brain TR=3132 ms). We acquired one fieldmap per subject per task with the identical volume and parameters of each EPI scan, and one five-minute magnetization-prepared rapid gradient-echo (MP-RAGE) T1-weighted 1 mm isotropic anatomical scan with whole-brain coverage (176 slices; slice thickness=1 mm; gap between slices=0.5 mm; TR=2730 ms; TE=3.57 ms; field of view=256 mm × 256 mm; matrix size=256 × 256; voxel size=1 × 1 × 1mm^3^ resolution).

For the emotion processing task, we used a 30° tilted sequence optimised to minimise dropout in the ventral prefrontal cortex and amygdalae (Nord et al., 2017b; Weiskopf et al., 2006). Note that our regions of interest (subgenual anterior cingulate cortex, sgACC, and amygdalae) show increased susceptibility artefacts (i.e. signal dropout) at higher field strengths, advantaging our (relatively) lower field strength.

EPI data were analysed using Statistical Parametric Mapping (SPM12; Wellcome Trust Centre for Neuroimaging, London, www.fil.ion.uck.ac.uk/spm; release date 1 Oct 2014) in MATLAB R2018a. After removing the first six volumes from each time series to allow for T1 equilibration, the remaining volumes were realigned to the seventh volume, coregistered to each subject's anatomical scan, normalized into standardized space (Montreal Neurological Institute template), and smoothed using an 8 mm full width at half maximum Gaussian kernel. Following realignment, all image sequences were examined for movements greater than 1.5 mm or rotations greater than 1° in any direction. No problematic images were identified; therefore, no images were removed and replaced using interpolation. Following normalization, anatomical images were manually checked for artefacts related to overfitting.

One n-back task scan (first-degree relative) was lost due to excessive motion (partly out of the field of view) and was therefore excluded from all n-back analyses (*N* = 98), but included in emotion processing analyses (*N* = 99).

In first-level analyses, regressors of interest were convolved with a synthetic hemodynamic response function time-locked to the onset of the corresponding event (emotion task: each 16-second block; n-back task, each 18-second block). We included six movement regressors of no interest in all subjects, and an error regressor of no interest for error trials in subjects who made gender discrimination errors on the emotion processing task. For both tasks, fixation periods constituted an implicit baseline. Using the general linear model, parameter estimate images were estimated for each regressor, and combined to create contrasts for each task (see Supplemental Table 1).

Second-level analyses were constructed using the standard summary statistics approach to random effects analysis. Our primary analysis examined differences between the groups in *a priori* regions-of-interest known to be activated by these tasks and implicated in dysfunctional cognitive/emotion processing in depression: each DLPFC for the n-back task; and sgACC and each amygdala for the emotional faces task. To identify the DLPFC, we used a 10mm-radius sphere centred on left DLPFC coordinates from a meta-analysis n-back activation in depression (−44,20,30) (Wang et al., 2015), and the corresponding coordinate in the right hemisphere (44,20,30). We used anatomical ROIs to identify the amygdalae (WFU Pickatlas, version 3.0.5) and sgACC (Nord et al., 2017b). See Supplemental Figure 2 for amygdala (2A) and sgACC (2B) ROIs.

#### 2.4.1 Co-primary analyses

We tested for group effects by extracting the average parameter estimate across all ROI voxels in each subject for our co-primary outcomes: in the emotion processing task, activation in the amygdala and sgACC in the fearful>neutral faces contrast; in the n-back task, activation in the DLPFC for the 3-back>1-back contrast. These three co-primary analyses tested the effect of group (depressed, low-risk, and first-degree relatives) on average ROI values using three mixed ANOVAs in SPSS 22.0 (IBM Comp, Armonk, NY). Significance is reported using Bonferonni correction for these three ROIs.

#### 2.4.2 Follow-up analyses

When there was a main effect of group in the primary analysis that survived Bonferroni correction for the three co-primary outcomes, we conducted post-hoc linear contrasts to illustrate the direction of effect between each pair of groups. We also conducted a supplemental analysis of the secondary contrast in the emotion processing task (happy>neutral), and sensitivity analyses to ensure that our primary result was not driven by differences in behavioural performance or history of antidepressant use, irrespective of whether the main effect was significant. We then conducted correlation analyses to assess whether our primary outcomes (i.e., ROI activation) were associated with depression (HAMD or BDI), anxiety (BAI), or anhedonia (SHAPS) symptoms within the depressed group alone (results reported using Bonferroni correction for the four symptom measures).

Finally, we performed correlations examining the relationships between activation within our ROIs *across* the tasks (specifically, the relationship between average DLPFC activation during the n-back task and amygdala activation/sgACC deactivation during the emotional face task; corrected for *N* = 4 comparisons, i.e. amygdala and sgACC activation for the fearful>neutral and happy>neutral contrasts).

#### 2.4.3 Exploratory whole-brain analyses

Whole-brain activation across all participants for each task is reported in Supplemental Figures 3 (n-back task) and 4 (emotion processing task). We applied a cluster-forming threshold of *p*<0.05 (FWE-corrected) and report *p*-values at the voxel- and cluster-corrected levels. For completeness, for each co-primary analysis, we also report the results of exploratory whole-brain one-way ANOVAs (*F*-tests) for the effect of group in SPM (cluster-forming threshold *p*<0.001 uncorrected), for each contrast in each task (see Supplemental Materials 4 (n-back) and 5 (emotion processing)). For these exploratory analyses we applied family-wise error (FWE) correction at the cluster level. We also report voxel-level activation within our *a priori* ROIs using small volume correction.

### 2.5 Power analyses

To determine our sample size, we ran power analyses for each region using G*Power 3.1.9.2 (ANOVA: fixed effects, omnibus, one-way). We expected a moderate-to-large effect size between the groups (Cohen's *d*~0.65): a previous study reported effect sizes of 0.58 and 0.83 for right and left amygdala, respectively, comparing low-risk controls and an at-risk sample during emotional face processing (Monk et al., 2008). With group sizes of *N* = 39, *N* = 30, and *N* = 30, we had 80% power to detect an effect size of f^2^~0.32 (moderate-to-large) in a one-way ANOVA.

For the correlation analyses with symptoms, a previous study found a large effect size (*r* = 0.63) for the relationship between amygdala responsivity and BDI scores in depressed patients (Hamilton and Gotlib, 2008). Assuming a moderate-to-large effect size of *r* = 0.45 (correlation: point biserial model), we required 33 subjects to achieve 80% power.

## 3 Results

### 3.1 Clinical, demographic and behavioural data

There were no differences across the three groups with respect to mean age (*F*(2.95)=1.890, *p* = 0.157) or the proportion of male and female participants (*X*^2^=0.734, *p* = 0.693). However, we included sex as a covariate in our analyses, due to strong evidence for gender differences in depression (Weissman and Klerman, 1985). We tested for the association between age and any measures of interest (i.e., behavioural and fMRI), and controlled for age when it was associated with a measure of interest. There were no differences between high-risk and low-risk groups on either clinical scales or reaction times (see Supplemental Materials 1).

There were no group differences on n-back behavioural performance (either using *d'* (*F*(2,94)=2.25, *p* = 0.111, *η_p_*^2^=0.046), or participants’ *d'* difference score (*F*(2,97)=1.26, *p* = 0.289, η_p_^2^=0.026); both analyses controlled for sex but not age, as neither *d'* nor *d'* difference scores were associated with age (*r*=−0.079, *p* = 0.439; *r* = 0.160, *p* = 0.116)). However, performance was quite variable on the n-back task (Table 1), and patients had numerically poorer *d'*; for this reason, we performed sensitivity fMRI analyses including the *d'* difference score (3-back *d'* – 1-back *d'*) as a covariate.

**Table 1:** Participant characteristics and behavioural performance.

	Controls	Relatives	Patients
N	30	30	39
% F	50	60	51
Age	32.10 (8.68)	28.67 (8.40)	33.38 (10.97)
FSIQ	110.54 (4.45)	109.73 (4.72)	107.30 (7.76)
HAM-D	1.17 (1.47)	2.40 (4.95)	21.64 (3.30) *
BDI	1.53 (2.15)	1.86 (3.09)	27.41 (6.76) *
SHAPS	5.37 (5.21)	5.07 (4.93)	18.97 (9.09) *
BAI	3.00 (4.21)	4.17 (5.84)	25.59 (12.69) *
Age onset	n/a	n/a	19.97 (9.09)
No. episodes	n/a	n/a	2.77 (1.63)
% first-degree relative w/ MDD	0	100	25.64
% attempted suicide	n/a	n/a	31
% past ADM	n/a	n/a	41
% past PT	n/a	n/a	64
% anxiety disorder	0	0	74.3
% past SD	0	0	17.9
% accuracy (emotion)	96.4 (1.0)	96.6 (0.6)	97.3(0.9)
d’	2.0 (0.9)	1.9 (1.0)	1.6 (0.9)
% hits: 3-back	65.2 (19.5)	59.6 (22.8)	54.4 (21.6)
% hits: 1-back	91.7 (13.6)	100 (0)	87.1 (22.9)

Figures represent means (SDs). F = female; FSIQ = Full Scale Intelligence Quotient; HAM-D = Hamilton Rating Scale for Depression; BDI = Beck Depression Inventory; BAI = Beck Anxiety Inventory; SHAPS = Snaith-Hamilton Pleasure Scale, note reverse scoring; No. = number; MDD = Major Depressive Disorder;% past ADM = per cent of patients with any previous history antidepressant medication use (no patients were currently medicated: see Methods);% past PT = per cent of patients with a history of psychological therapy;% anxiety disorder = per cent of patients meeting criteria for an Axis I anxiety disorder;% SD = per cent meeting criteria for past substance abuse or dependence (restricted to depressed episodes as an inclusion criteria);% accuracy/hits = per cent accuracy at the gender classification task on the emotion processing paradigm; d’ (our primary measure of performance on the n-back, see text), and per cent hits on the 3-back and 1-back conditions of the n-back task. Note the substantially better performance on the 1-back condition across all three groups.*F-test *p*<0.05 for effect of group.

There were no group differences in accuracy on the emotion processing task (see Table 1; accuracy analysed using a Kruskal-Wallis test due to non-normality: *X^2^*(2)=4.57, *p* = 0.102, *η_p_*^2^=0.044).

### 3.2 fMRI results

Across all participants, we found bilateral DLPFC activation during the n-back task (Supplemental Materials 2; Supplemental Table 3), activation of the amygdalae in the emotion processing task (Supplemental Materials 3; Supplemental Table 5), and sgACC deactivation in the emotion processing task (Supplemental Materials 3; Supplemental Table 5). See Supplemental Figure 3 for whole brain activation within each group during the n-back task, Supplemental Figures 4 and 5 for whole brain activation within each group for each emotion contrast, and Supplemental Table 6 for co-primary outcome measures (mean, standard deviation, effect sizes, and one-sample t-statistics) for each group separately.

#### 3.2.1 Co-primary analysis: group differences in DLPFC activation during working memory

We did not find an association between age and DLPFC activation (left: *r*=−0.137, *p* = 0.177; right: *r*=−0.141, *p* = 0.165; Spearman's rho due to the non-Gaussian distribution of age in our sample). Therefore we did not include age in this model.

**Fig. 1:**
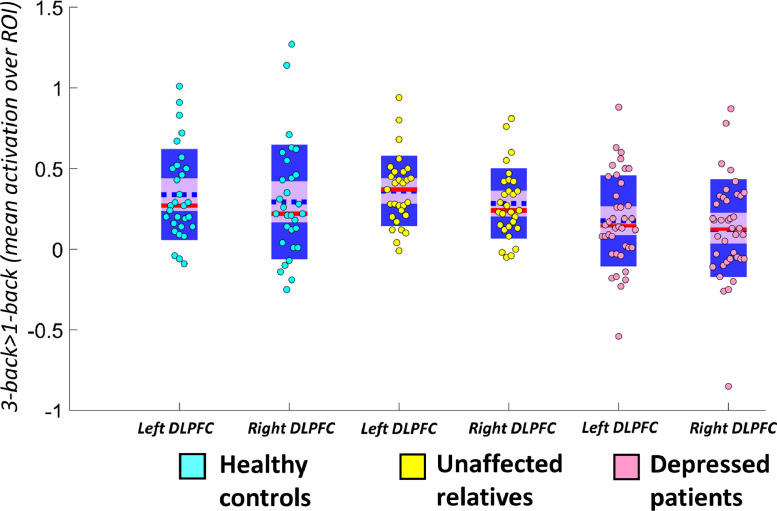
Distribution and summary statistics of parameter estimates in the left and right DLPFC ROIs and whole-brain analysis results. The blue dotted line represents the mean; the red line represents the median; light purple patch shows the 95% confidence interval; darker purple patch shows the standard deviation of the mean. In the full ANOVA, there was a significant main effect of group (overall group effect *p* = 0.012) and laterality (*p* = 0.006). Depressed patients had significantly lower DLPFC activation compared to both unaffected relatives (*mean difference*=0.169, *p* = 0.012, Cohen's *d* = 0.674) and low-risk controls (*mean difference*=0.162, *p* = 0.014, Cohen's *d* = 0.560) (both significant at corrected threshold of *p* = 0.0167), and there were no differences in DLPFC activation between controls and unaffected relatives (*mean difference*=0.007, *p* = 0.926, Cohen's *d* = 0.01). DLPFC=dorsolateral prefrontal cortex; ROI=region of interest.

Including sex as a covariate, we found a significant effect of group on DLPFC activation (*F*(2,92)=4.654, *p* = 0.012, η_p_^2^=0.092), stronger activation in the left than the right DLPFC (*F*(1,92)=8.042, *p* = 0.006, η_p_^2^=0.080), but no laterality-by-group interaction (*F*(2,92)=0.347, *p* = 0.708) (see Fig. 1). The main effect of group survived Bonferroni correction for the three co-primary analyses (corrected threshold: *p* = 0.0167).

#### 3.2.2 Follow-up analyses: n-back task

To clarify the main effect of group found in our primary analysis, we computed post-hoc analyses (least-squared difference (LSD) tests), which revealed that patients had significantly lower DLPFC activation compared to both unaffected relatives (*mean difference*=0.169, *p* = 0.012, Cohen's *d* = 0.674) and low-risk controls (*mean difference*=0.162, *p* = 0.014, Cohen's *d* = 0.560). There were no differences in DLPFC activation between controls and unaffected relatives (*mean difference*=0.007, *p* = 0.926, Cohen's *d* = 0.01). The difference between patients and relatives and the difference between patients and low-risk controls both survived Bonferroni correction for the three co-primary outcomes (corrected threshold: *p* = 0.0167).

Next, we tested whether this effect was driven by differences in n-back performance between the groups. The main effect of group on DLPFC activation remained significant when including n-back performance as a covariate (*F*(1,91)=4.37, *p* = 0.015, η_p_^2^=0.088). There was also no correlation between DLPFC activation and n-back performance, either overall (*r*=−0.108, *p* = 0.290), or within any group (low-risk controls: *r*=−0.030, *p* = 0.876; unaffected relatives: *r* = 0.035, *p* = 0.860; depressed patients: *r*=−0.166, *p* = 0.320; all analyses controlled for sex). We then tested for the possible impact of prior antidepressant use in the depressed group. There was no effect of past antidepressant use on DLPFC activation (*t*(1,27.43)=0.252, *p* = 0.803).

We found no associations between DLPFC activation and questionnaire measures of symptoms in depressed patients (BDI: *r*=−0.114, *p* = 0.491; SHAPS: *r*=−0.027, *p* = 0.869; BAI: *r* = 0.082, *p* = 0.619; HAMD *r*=−0.114, *p* = 0.491). There were also no associations between symptom measures and n-back task performance (*d'* difference) within the patient group (BDI: *r* = 0.147, *p* = 0.343; SHAPS: *r* = 0.174, *p* = 0.290; BAI: *r*=−0.085, *p* = 0.608; HAMD *r* = 0.179, *p* = 0.274).

#### 3.2.3 Exploratory whole-brain analysis: n-back task

We conducted an exploratory *F-*test of the effect of group on whole-brain activation for the 3-back>1-back contrast. There was no FWE-significant effect of group on whole-brain activation (see Supplemental Materials 4 and Supplemental Tables 7 and 8).

#### 3.2.4 Co-primary analysis: group differences in amygdala activation during fear processing

We conducted a repeated-measures ANOVA, with within-subjects factor laterality, and a between-subjects factor of group. As with the n-back analysis, we included sex as a covariate; in this model, we also included age as a covariate because the association between age and fearful>neutral contrast for the right amygdala was significant (*r*=−0.232, *p* = 0.021).

**Fig. 2:**
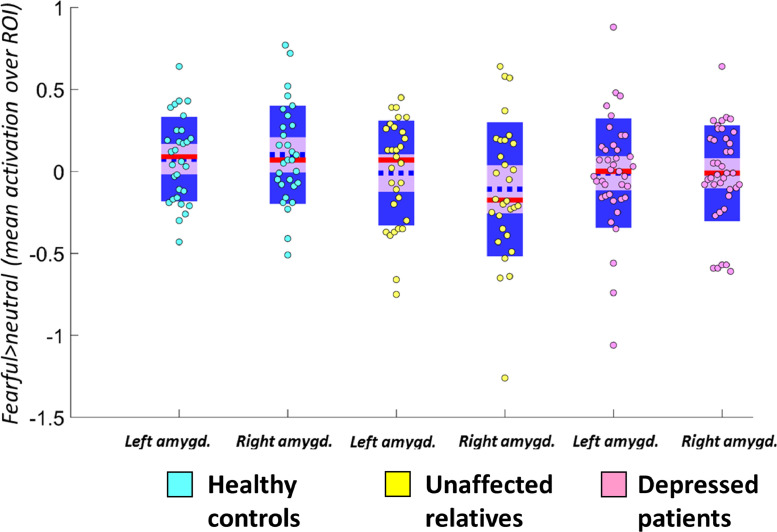
Distribution and summary statistics of parameter estimates in the left and right amygdala ROIs for the primary contrast (*fearful>*neutral) for each group. The blue dotted line represents the mean; the red line represents the median; light purple patch shows the 95% confidence interval; darker purple patch shows the standard deviation of the mean. ROI=region of interest; amygd=amygdala.

We did not find a significant effect of group (*F*(2,92)=0.616, *p* = 0.542), an interaction between group and laterality (*F*(2,92)=0.241, *p* = 0.786), or an effect of laterality (*F*(1,92)=0.016, *p* = 0.901) (see Fig. 2).

We did not find any associations between amygdala activation during fearful emotion processing and questionnaire measures in depressed patients for the amygdala during fearful>neutral (BDI: *r*=−0.182, *p* = 0.266; SHAPS: *r*=−0.266, *p* = 0.167; BAI: *r* = 0.015, *p* = 0.930; HAMD *r*=−0.299, *p* = 0.064) or happy>neutral contrasts (BDI: *r*=−0.210, *p* = 0.199; SHAPS: *r*=−0.222, *p* = 0.174; BAI: *r*=−0.022, *p* = 0.895; HAMD *r*=−0.239, *p* = 0.143)

#### 3.2.5 Secondary analyses: group differences in amygdala activation during happy processing

For our secondary contrast (happy>neutral faces), we again conducted a repeated-measures ANOVA with within-subjects factor laterality and a between-subjects factor of group, including sex and age as covariates because the association between age and happy>neutral contrast activation for the right amygdala was significant (*r*=−0.212, *p* = 0.035).

We did not find a significant effect of group (*F*(2,92)=2.87, *p* = 0.062), an interaction between group and laterality (*F*(2,92)=1.23, *p* = 0.296), or an effect of laterality (*F*(2,92)=1.26, *p* = 0.265).

Within the depressed group alone, there was no effect of past antidepressant use on amygdala activation during either fearful>neutral (*t*(1,37)=0.113, *p* = 0.911) or happy>neutral face processing (*t*(1,37)=0.384, *p* = 0.703).

#### 3.2.6 Co-primary analysis: group differences in sgACC deactivation during fear processing

**Fig. 3:**
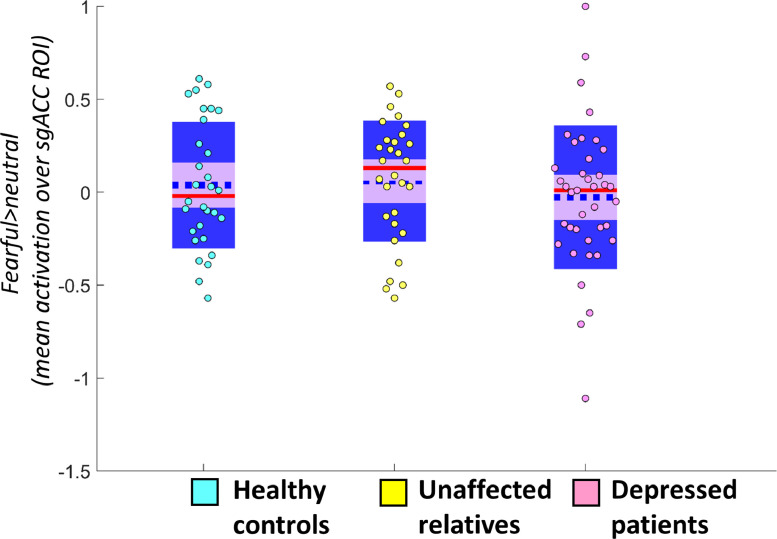
Distribution and summary statistics of parameter estimates in the sgACC ROI for the primary contrast (*fearful>*neutral) for each group. The blue dotted line represents the mean; the red line represents the median; light purple patch shows the 95% confidence interval; darker purple patch shows the standard deviation of the mean. ROI=region of interest; sgACC=subgenual anterior cingulate cortex.

As in the DLPFC and amygdala models, we included sex as a covariate in our primary analysis testing for the effect of group on sgACC deactivation. We did not include age as a covariate because age was not significantly associated with sgACC activation during fearful emotion processing (*r*=−0.141, *p* = 0.164). We found no significant main effect of group (*F*(2,98)=0.202 *p* = 0.818) (see Fig. 3).

For sgACC deactivation during fearful>neutral faces, neither HAM-D nor BDI scores correlated significantly with deactivation, though both showed a trend in the same direction: greater sgACC deactivation was associated with higher levels of depression (HAM-D (*r*=−0.284, *p* = 0.080); BDI (*r*=−0.302, *p* = 0.062)). There were no associations between sgACC deactivation during happy emotion processing and anxiety (BAI; *r* = 0.028, *p* = 0.865) or anhedonia (SHAPS): *r*=−0.126, *p* = 0.443) scores; nor between sgACC deactivation during fearful emotion processing and anxiety (BAI: *r*=−0.062, *p* = 0.709) or anhedonia (SHAPS: *r*=−0.073, *p* = 0.659) scores.

#### 3.2.7 Secondary analyses: sgACC activation during happy processing

For our secondary contrast (happy>neutral faces), we again conducted a repeated-measures ANOVA with within-subjects factor laterality and a between-subjects factor of group, including sex and age as covariates because the association between age and happy>neutral contrast activation for the sgACC was significant (*r*=−0.244, *p* = 0.015). We did not find a significant effect of group (*F*(2,98)=0.191, *p* = 0.827).

**Fig. 4:**
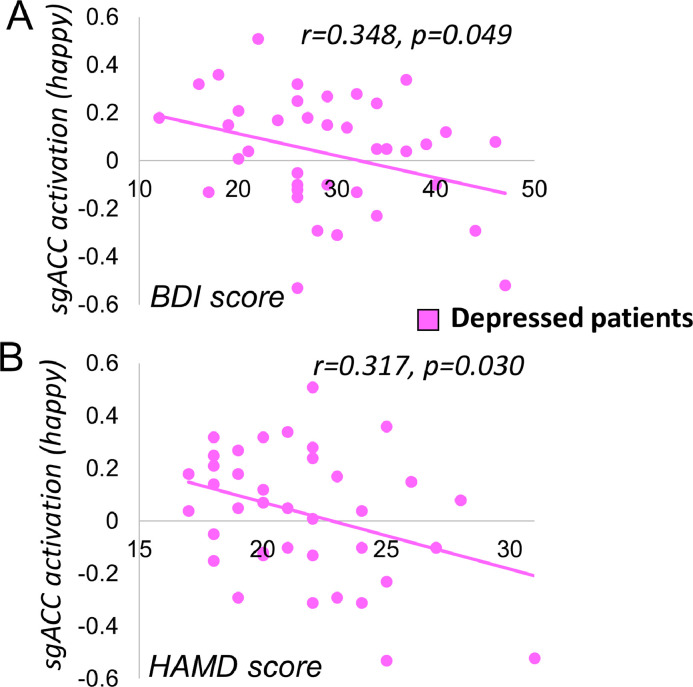
Association between sgACC deactivation and depression measures. Relationship between subgenual anterior cingulate cortex (sgACC) deactivation to happy vs neutral faces and symptom scores in depressed patients for Beck Depression Inventory (BDI, *p* = 0.049, non-significant at corrected threshold of *p* = 0.0063, A) and Hamilton Depression rating scale (HAM-D, *p* = 0.030, non-significant at corrected threshold of *p* = 0.0063, B).

Again, within the depressed group alone, there was no effect of past antidepressant use on sgACC deactivation during either fearful>neutral (*t*(1,37)=0.144, *p* = 0.886) or happy>neutral face processing (*t*(1,37)=0.221, *p* = 0.827). sgACC deactivation to happy>neutral faces positively correlated with both measures of depression: HAM-D (*r*=−0.348, *p* = 0.030) and BDI (*r*=−0.317, *p* = 0.049) (see Fig. 4), indicating that sgACC deactivation was associated with higher levels of depressive symptoms. Neither of these associations achieved significance at the Bonferroni-corrected threshold of *p* = 0.0063.

#### 3.2.8 Exploratory whole-brain analysis: emotion processing task

We conducted an exploratory three-way *F-*test of the effect of group on whole-brain activation in our primary (fearful>neutral) and secondary (happy>neutral) contrasts. We found no FWE-significant effect of group on whole-brain activation (see Supplemental Materials 5 and Supplemental Table 9).

#### 3.2.9 Correlations across emotion processing and n-back tasks

**Fig. 5:**
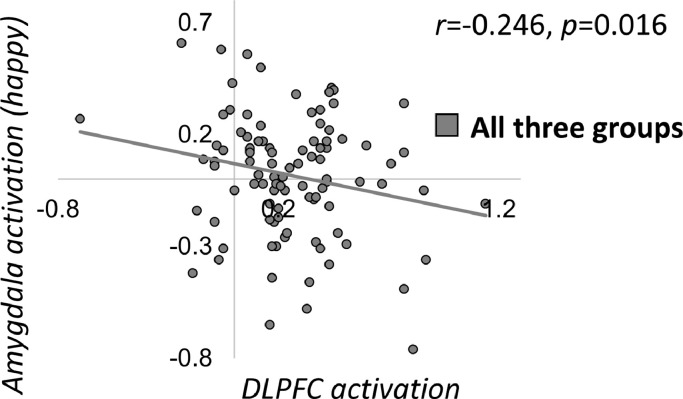
Relationship between amygdala activation to happy faces (average across left and right) and average dorsolateral prefrontal cortex (DLPFC) activation during the n-back task (*r*=−0.246, *p* = 0.016, non-significant at corrected threshold of *p* = 0.0125). DLPFC=dorsolateral prefrontal cortex.

Motivated by the cognitive neuropsychological hypothesis that the DLPFC also influences bottom-up emotional responses (Roiser and Sahakian, 2013), we also tested whether there was an association between DLPFC activation during the n-back task and activation of the amygdala (collapsed across left and right) or sgACC deactivation, for both fearful and happy faces (across all participants *N* = 98; 4 correlations). There were nominally significant negative relationships between DLPFC activation and both amygdala/sgACC activation during happy emotion processing (amygdala: *r*=−0.246, *p* = 0.016; sgACC: *r*=−0.202, *p* = 0.049), such that participants with the highest DLPFC activation had the lowest amygdala and sgACC activation (see Fig. 5). These analyses also controlled for age, due to relationships between the variables of interest and age. There was no association between performance on the n-back task (*d'*) and sgACC deactivation, either during fearful (*r* = 0.005, *p* = 0.965) or happy emotion processing (*r* = 0.119, *p* = 0.248).

However, neither of the above associations survived Bonferroni correction for four tests (threshold: *p* = 0.0125). There was also no significant relationship between DLPFC activation during the n-back task and either amygdala or sgACC activation during fearful emotion processing (*r*=−0.115, *p* = 0.263 and *r*=−0.012, *p* = 0.904, respectively; all of the above analyses controlled for sex).

### 4 Discussion

We compared neural activation during emotion processing (“hot” cognition) and executive function (“cold” cognition) in unmedicated patients with MDD, unaffected first-degree relatives of depressed individuals, and low-risk controls. This study represents a direct test of the predictions of the cognitive neuropsychological model; namely, that risk for depression would be associated with aberrant “hot” cognitive processing, but resilience against depression (i.e., euthymia) would be associated with intact executive function.

We measured dorsal prefrontal activation (associated with ‘cold’ cognition) using an n-back working memory task, and ventral prefrontal/subcortical (associated with ‘hot’ cognition) using an incidental emotion task. We found that unaffected first-degree relatives showed indistinguishable DLPFC activation from low-risk controls during working memory, while depressed patients showed hypoactivation in the DLPFC, compared with low-risk controls. We did not detect group differences in our *a priori* ROI analysis of the emotion processing task. Our findings lend preliminary support to one central prediction of the cognitive neuropsychological model: the hypothesis that resilience to depression may be mediated by executive function networks. However, we did not find clear support for the second prediction of the model: that risk may be conferred through negatively biased emotion processing.

Our finding of disrupted DLPFC activation in depressed patients supports a large literature of DLPFC abnormalities in depression (Baxter et al., 1989; Bench et al., 1993; Gärtner et al., 2018; Korgaonkar et al., 2013; Pu et al., 2011; Siegle et al., 2007; Wang et al., 2015), though note hyper-activation is often reported for easier n-back conditions. Two other studies have measured DLPFC activation during working memory in participants with high familial risk of depression (Mannie et al., 2010; Watters et al., 2019). Whilst one reported greater activation during working memory in high-risk participants (Mannie et al., 2010), a well-powered recent study, with some differences in design, reported hypoactivation (Watters et al., 2019). In both studies, high-risk participants had higher levels of depression and anxiety symptoms (compared to low-risk controls; note that in the more recent study, inclusion of mood as a covariate did not alter the finding of hypoactivation). Nevertheless, our sample of high-risk participants showed statistically indistinguishable ratings of depression, anxiety, and anhedonia, compared to low-risk controls, and equivalent accuracy on the n-back task. Therefore, the lack of differences in DLPFC activation compared to low-risk controls we report could result from sampling differences compared to the previous two studies (i.e., our population might consist of more resilient individuals, defining resilience as an absence of depressive symptoms).

The age range we sampled from might support this speculation: our first-degree relatives were on average 15 years older than the mean age of depression onset (Lewinsohn et al., 1994), suggesting that at least a subset may be past the period of highest risk for developing depression (although first-degree relatives of depressed patients remain at increased risk of developing MDD across the lifespan (Williamson et al., 1995)). This is also consistent with a recent report that participants with high polygenic risk for depression showed lower activation in fronto-parietal regions during the n-back than those with low polygenic risk (Yüksel et al., 2017).

It is also possible to interpret DLPFC hypoactivation in the depressed group as a state-dependant alteration. Distinguishing between this interpretation and one of ‘resilience’ is impossible given the design of our study, which did not include euthymic depressed patients. To better characterise the role of the DLPFC in risk and resilience for depression, future studies could track depressed patients longitudinally to assess whether activation normalises following recovery. However, in a previous study, euthymic MDD patients showed comparable lateral prefrontal activation during working memory to healthy controls (Schöning et al., 2009), and pharmacological (Brody et al., 2001; Fales et al., 2009), psychological (Brody et al., 2001; Goldapple et al., 2004), and somatic treatments for depression (Perrin et al., 2012) have all been found to normalise DLPFC activation. Similarly, most non-invasive brain stimulation treatments for depression target the DLPFC (Blumberger et al., 2018; George et al., 2000; Loo et al., 2012; Nord et al., 2019; Nord and Roiser, 2015), and there is preliminary evidence that DLPFC activation may be a ‘biomarker’ for treatment response to these interventions (Nord et al., 2019; Weiduschat and Dubin, 2013).

We did not find any group differences in sgACC or amygdala responsivity to emotional faces, either in first-degree relatives or currently-depressed patients. Some previous studies have shown aberrant neural activation during emotion processing in first-degree relatives, compared to low-risk controls (Chan et al., 2009; Monk et al., 2008), while others have not (Mannie et al., 2011). Heterogeneity within the patient sample may have obscured differences: we found that sgACC deactivation to happy faces was associated with higher depression scores, on both observer-rated (HAM-D) and self-report (BDI) measures in the patient group, although this did not survive stringent correction for multiple comparisons.

Although we investigated executive function and emotion processing separately, these mechanisms interact strongly in the aetiology of depression. Emotional reactivity (and corresponding limbic circuit abnormalities) could in part originate through inefficient prefrontal regulatory mechanisms; amygdala hyper-activation has previously been associated with lower DLPFC activation (Siegle et al., 2007), and many studies have reported differing effects of depression on working memory for neutral versus valences emotional stimuli (Berman et al., 2011; Bertocci et al., 2012; Kerestes et al., 2012; Schweizer et al., 2018).

## 5 Limitations

Our null findings in the emotion processing paradigm may be reflective of the task we chose (incidental emotion processing); the n-back task (and DLPFC) might have been more sensitive to group differences. There is evidence that amygdala and sgACC responsivity during emotion processing tasks has poor within-subject reliability (Nord et al., 2017b), while moderate-to-good reliability has been reported for DLPFC activation during the n-back task (Plichta et al., 2012). Importantly, for the emotion processing task, we only detected significant activation in the amygdala for the fearful>neutral faces contrast, limiting the interpretability of the null effect of group on sgACC activation or on our secondary contrast (happy>neutral). It will also be essential to probe these circuits using other paradigms, in particular reward processing. There is a literature suggesting that reward processing in at-risk individuals shows a depression-like pattern: diminished orbitofrontal cortex (OFC) responses during reward delivery (McCabe et al., 2012), and greater OFC activation to aversive outcomes (McCabe et al., 2012) and omitted rewards (Macoveanu et al., 2014). Most convincingly, a longitudinal study revealed that never-depressed adolescents who later developed depressive symptoms had lower ventral striatum responses during reward anticipation than those who showed no symptoms at either time-point (Stringaris et al., 2015). In addition, it is a limitation of our study that we did not measure individual differences in ratings of emotional faces; this may have yielded insight into our lack of findings in this task. We also did not measure or endeavour to match groups on socioeconomic status or ethnicity, a limitation of our sample characterisation.

Our power analyses indicated that our sample size (*N* = 99) was sufficient to detect a moderate-to-large effect of group. However, a larger sample size would be required to detect more subtle relationships between brain activation and symptom measures. In addition, it would be useful in future to better characterize the true risk level of our first-degree relative group: without a longitudinal design, it is impossible to know which individuals were at risk, and which were resilient, which limits the interpretation of our findings.

## 6 Conclusion

Our study was an integrative attempt to directly test the predictions of the cognitive neuropsychological model of depression by measuring neural activation during “hot” and “cold” cognition in individuals with a high and low familial risk of depression, compared to patients with current MDD. While some studies have tested aspects of this hypothesis separately (e.g., measuring dorsal prefrontal activation during “cold” cognition in an at-risk group compared to controls (Watters et al., 2019)), our study tested both neural mechanisms in all three groups. ‘Hot’ cognitive processing was indistinguishable between groups. Moreover, we found no evidence for differences in DLPFC function in never-depressed participants with a family history of depression, compared to low-risk controls. Patients with major depression showed substantial DLPFC hypoactivation during a difficult working memory task, compared to both high- and low-risk individuals.

This could have implications not only for understanding the neurobiology of family risk for depression, but also potentially in developing treatments that target prefrontal mechanisms for currently-depressed patients. Improving executive function might represent one way of treating or preventing depression, through top-down control of emotional processing regions: several trials found (unexpectedly) that cognitive training in dementia improved depressive symptoms (Sitzer et al., 2006). An alternative approach involves directly targeting prefrontal mechanisms in depression with non-invasive brain stimulation: transcranial magnetic stimulation (George et al., 2000) and transcranial direct current stimulation (Loo et al., 2012; Nord and Roiser, 2015) have both shown efficacy at treating depression; the latter in particular may target executive function mechanisms (Nord et al., 2017a, 2019). Future work needs to better clarify the interaction between dorsal prefrontal and ventral prefrontal/subcortical responses in individuals at a familial risk for depression, with the view to preventing at-risk populations from developing depression, and better treating those who do.

## Role of the funding source

The funding source had no role in data collection, analysis, or write-up.

## Institutional board review

The study was approved by the London Queen Square NHS Research Ethics Committee (ID: 13/LO/1028).

## CRediT authorship contribution statement

**Nord CL:** Conceptualization, Methodology, Data curation, Resources, Formal analysis, Writing - original draft, Writing - review & editing. **Halahakoon DC:** Methodology, Data curation, Writing - review & editing. **Lally N:** Methodology, Data curation, Writing - review & editing. **Limbachya T:** Resources, Writing - review & editing. **Pilling S:** Resources, Writing - review & editing. **Roiser JP:** Conceptualization, Formal analysis, Writing - original draft, Writing - review & editing.

## Conflict of interest

JPR consults for Cambridge Cognition, Takeda Ltd and GE. The other authors report no conflict of interest.

## Appendix Supplementary materials

Image, application 1

Image, application 2
