# Supplementary material for: The neural basis of hot and cold cognition in depressed patients, unaffected relatives, and low-risk healthy controls: An fMRI investigation
Source: J Affect Disord. 2020 Sep 1;274:389–98. doi: 10.1016/j.jad.2020.05.022 (PMC7369634; doi:10.1016/j.jad.2020.05.022)
Supplement: Supplementary file 2 [file mmc2.docx]

Supplemental Materials

|  | N-back task | Emotion processing task |
| --- | --- | --- |
| Task duration (min:sec per run) | 9:33 | 6:38 |
| No. volumes | 183 | 127 |
| Task design | Blocked | Blocked |
| Regressors of interest | 3-back; 1-back | Happy; fearful; neutral |
| Regressors of no interest | 6 movement parameters | 6 movement parameters + errors |
| Contrasts of interest | 3-back > 1 back (co-primary) | fearful > neutral (co-primary)  happy > neutral (secondary) |
|  |  | faces > fixation (supplemental) |

**Supplemental Table 1.** *Characteristics of each task*

**
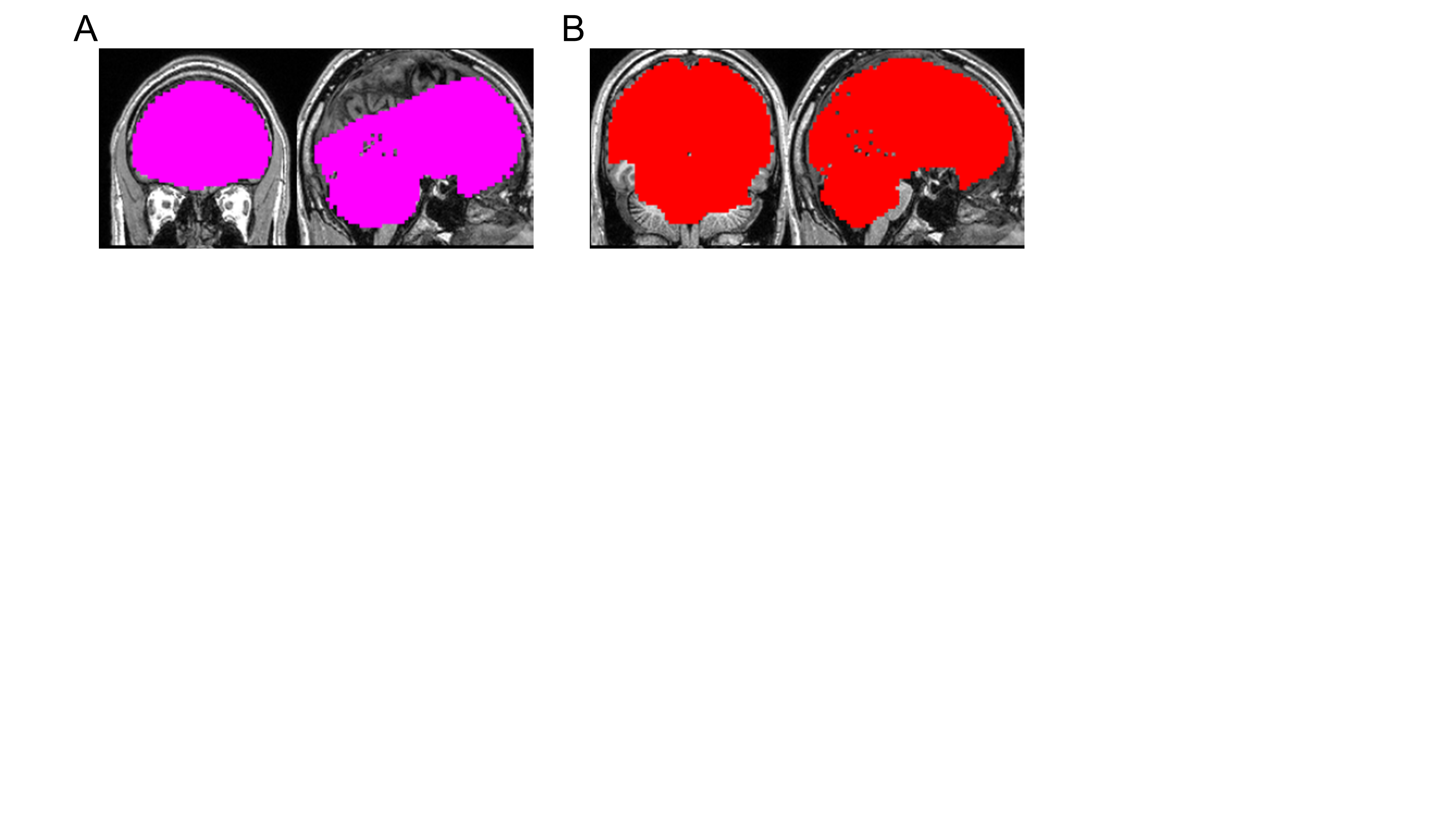
**

**Supplemental Figure 1. Whole-brain activation by group.** Second-level masks (overlaid on an example subject anatomical scan) for the emotion processing (A) and n-back (B) task. Note the slice thickness was 0.5mm greater for the n-back task (B), giving us fuller coverage.

**
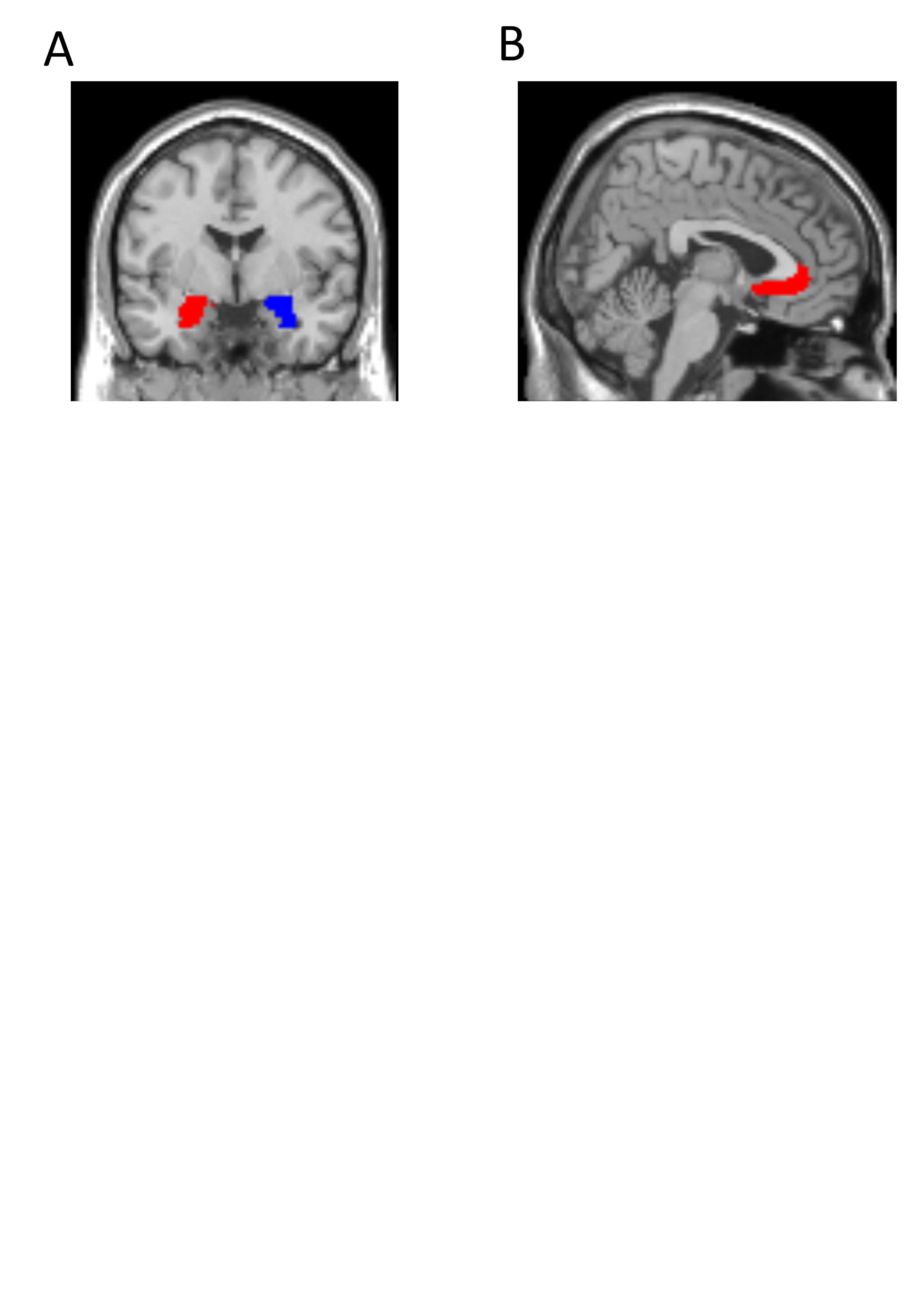
**

**Supplemental Figure 2.** Regions-of-interest (ROIs) used for the emotion processing task: left and right amygdala (A, from WFU Pickatlas, version 3.0.5) and a custom-made subgenual anterior cingulate cortex (B) (n-back ROI not shown, since it involved a 4mm-sphere surrounding a peak coordinate from a previous meta-analysis).


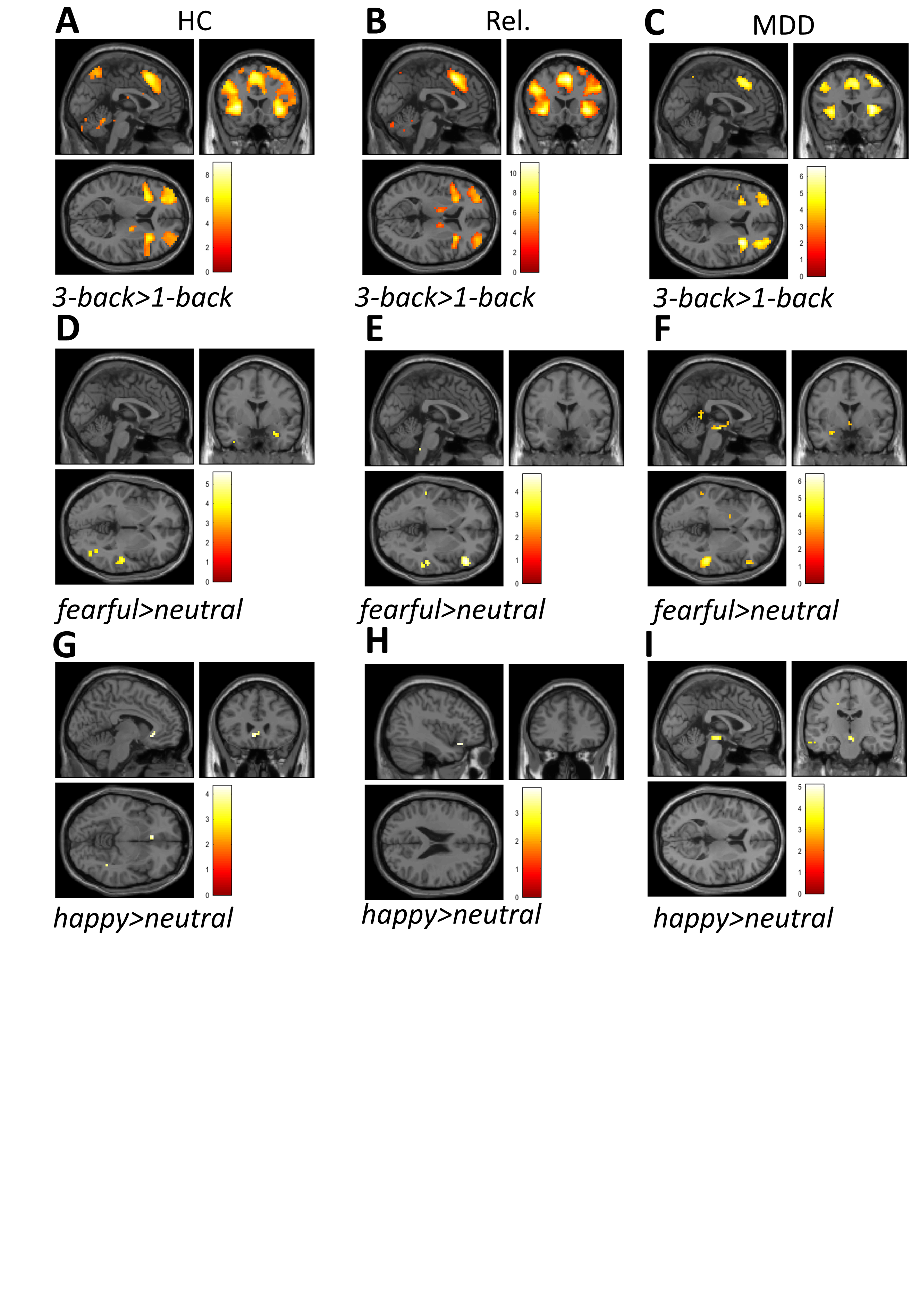


**Supplemental Figure 3. Whole-brain activation by group: n-back task.** HC= healthy controls; Rel.= unaffected first-degree relatives of patients with depression; MDD = major depressive disorder.

*
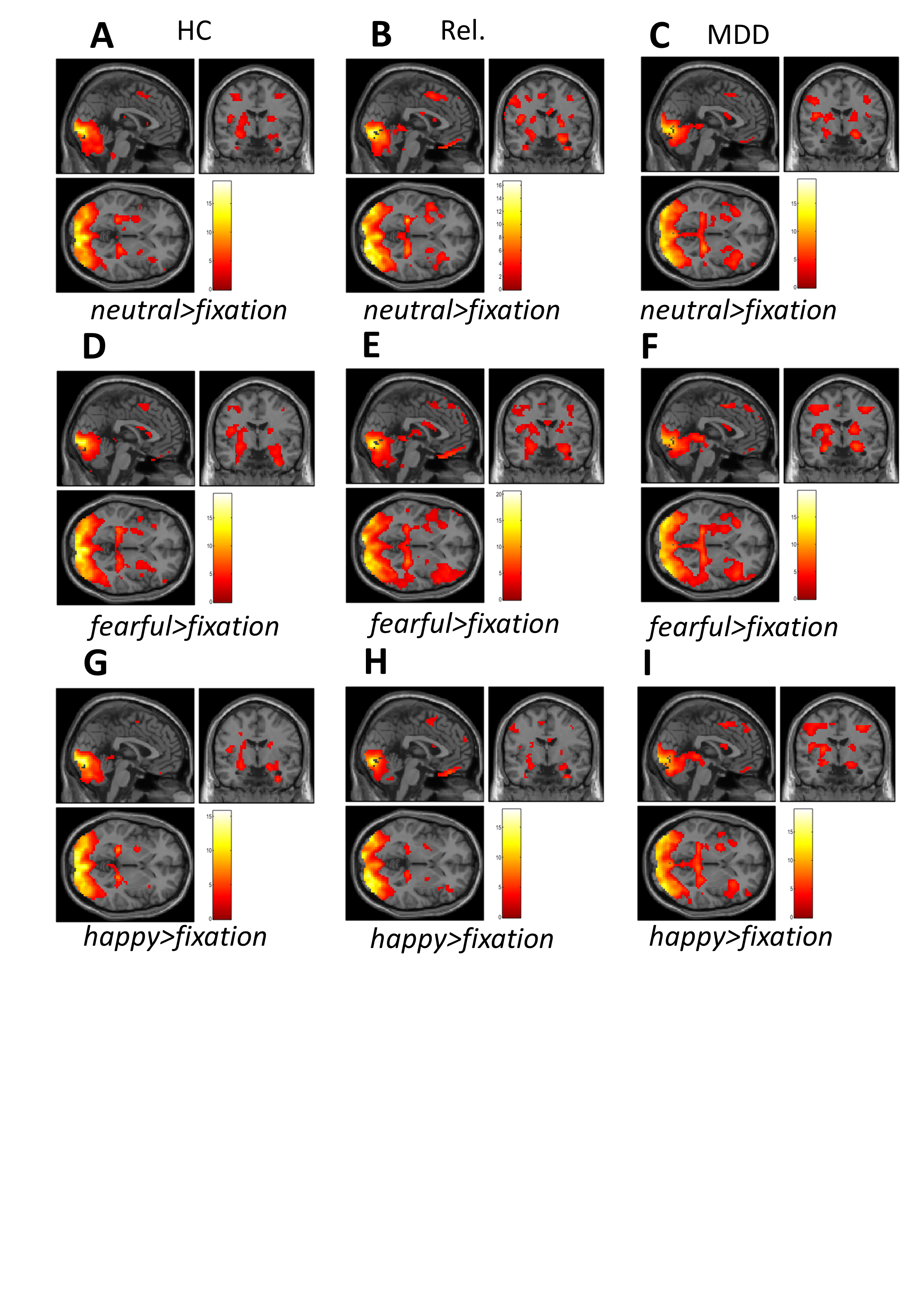
*

**Supplemental Figure 4. Whole-brain activation for contrasts (fixation baseline) by group.** Split by group and emotion>fixation contrast: neutral>fixation (A-C); fearful>fixation (D-F); happy>fixation (G-I). HC= healthy controls; Rel.= unaffected first-degree relatives of patients with depression; MDD = major depressive disorder. Note amygdala activation in coronal slices; sgACC activation observed in reverse contrast (data not shown).

**
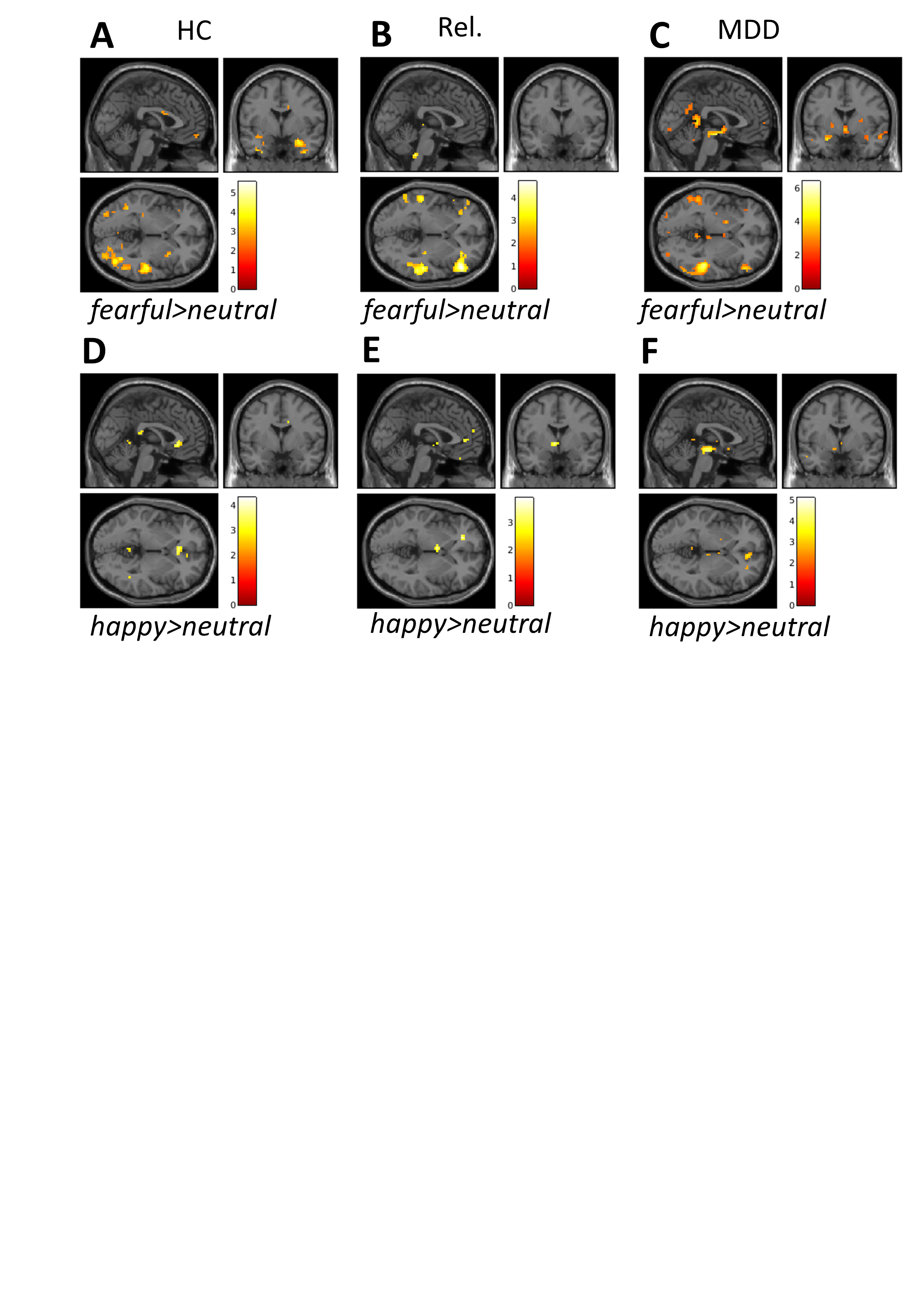
**

**Supplemental Figure 5. Whole-brain activation for contrasts (neutral faces baseline) by group.** Split by group and emotion contrast: our primary contrast, fearful>neutral (A-C); and our secondary contrast, happy>neutral (D-F), for healthy controls (=HC, in A and D), unaffected first-degree relatives of patients with depression (=Rel., in B and E), and patients with major depressive disorder (=MDD, in C and F). Image thresholded at *p*=0.01 (uncorrected) for visualisation purposes only.

1. Clinical, demographic, and reaction time analyses

In one-way ANOVAs with group as the between-subjects factor, there was no effect of group on age or FSIQ (both *p*>0.1); a chi-square test also revealed no effect of group on sex (*X*^2^=0.734, *p*=0.693). There were significant effects of group on HAM-D (*F*(2,96)=828.19, *p*<0.001), BDI (*F*(2,96)=346.61, *p*<0.001), SHAPS (*F*(2,96)=64.01, *p*<0.001), and BAI scores (*F*(2,96)=72.55, *p*<0.001). Post-hoc linear comparisons showed that healthy controls did not differ significantly from first-degree relatives on any measure (all *p*>0.05), but differed from depressed patients on all clinical scales: HAM-D (*t*(67)=20.47, *p*<0.001), BDI (*t*(67)=25.88, *p*<0.001), BAI (*t*(67)=22.59, *p*<0.001), and SHAPS (*t*(67)=13.61, *p*<0.001). First-degree relatives also differed from depressed patients on all clinical measures (HAM-D (*t*(67)=30.20, *p*<0.001), BDI (*t*(67)=19.06, *p*<0.001), BAI (*t*(67)=8.60, *p*<0.001), and SHAPS (*t*(67)=9.25, *p*<0.001)).

For both tasks, reaction time data conformed to assumptions of normality (Komolgorov-Smirnov tests, *p*>0.05). To test for an effect of group on n-back reaction times (correct responses only), we conducted a one-way ANOVA, finding no effect of group (*F*(2,94)=1.64, *p*=0.200, η_p_^2^=0.034). For the emotion processing task, we conducted a repeated-measures ANOVA with the within-subjects factor emotion (happy, fearful, or neutral faces), finding no main effect of emotion (*F*(2,192)=0.768, *p*=0.466, η_p_^2^=0.008), interaction with group (*F*(4,192)=0.564, *p*=0.689, η_p_^2^=0.012) or effect of group (*F*(2,96)=1.35, *p*=0.263, η_p_^2^=0.027).

1. Activation across groups: n-back task

The effect of high vs low working memory condition (3-back versus 1-back; whole-brain p<0.05, voxel-level FWE-corrected) evoked very large clusters of activation extending from a peak in the insula to the bilateral DLPFC, as well as several significant clusters elsewhere, including a large cluster with a peak in the posterior parietal cortex (see Supplemental Table 2). The inverse contrast evoked substantial VMPFC and posterior cingulate activation. An anatomical mask including Brodmann areas 9 and 46 confirmed substantial activation in the left and right DLPFC in the 3-back<1-back contrast (see Supplemental Table 3).

| Contrast | *p* (cluster-corrected) | Extent | *p* (voxel-corrected) | t(97) | X | Y | Z | region |
| --- | --- | --- | --- | --- | --- | --- | --- | --- |
| 3-back > 1-back | <0.001 | 4561 | <0.001 | 12.36 | 33 | 20 | -1 | insula, DLPFC |
|  | <0.001 | 289 | <0.001 | 12.24 | 30 | -64 | -31 | R cerebellum |
|  | <0.001 | 2073 | <0.001 | 10.99 | 42 | -43 | 44 | R PPC |
|  | 0.001 | 17 | <0.001 | 7.54 | 57 | -52 | -10 | R fusiform |
|  | <0.001 | 63 | <0.001 | 6.52 | -12 | -4 | 2 | L thalamus |
|  | <0.001 | 51 | <0.001 | 6.28 | 15 | -4 | -1 | R thalamus |
|  | 0.017 | 2 | 0.002 | 5.70 | 3 | 14 | 23 | R mid-cingulate |
|  | 0.017 | 2 | 0.009 | 5.29 | 0 | -52 | -16 | cerebellum |
|  | 0.026 | 1 | 0.014 | 5.18 | -21 | 47 | -10 | L VMPFC |
|  | 0.026 | 1 | 0.030 | 4.97 | 57 | -34 | -13 | lat. temporal |
| 1-back > 3-back | <0.001 | 1430 | <0.001 | 13.20 | -3 | 29 | -19 | L VMPFC |
|  | <0.001 | 8026 | <0.001 | 11.30 | -9 | -52 | 11 | L posterior cingulate cortex |
|  | 0.013 | 3 | 0.006 | 5.39 | 54 | 35 | 2 | R VLPFC |
|  | 0.026 | 1 | 0.030 | 4.97 | 24 | 11 | -10 | R putamen |
|  | 0.026 | 1 | 0.032 | 4.94 | 15 | -28 | -1 | R thalamus |
|  | 0.026 | 1 | 0.045 | 4.85 | 12 | -31 | 5 | R thalamus |

**Supplemental Table 2. Whole brain activation results: n-back task.** Whole-brain significant activation during n-back task, for the 3-back>1-back contrast and its inverse, 1-back>3-back (cluster-forming threshold *p*<0.05, FWE corrected at the voxel-level). BA=Brodmann area; L=left; R=right. DLPFC=dorsolateral prefrontal cortex; PPC=posterior parietal cortex; VMPFC=ventromedial prefrontal cortex; VLPFC=ventrolateral prefrontal cortex; lat.=lateral. Note, here and in Supplemental Table 3, that since we applied *p*<0.05 (FWE-corrected) as the cluster-forming threshold, all voxels surviving this automatically survive whole-brain correction in these results tables.

| Contrast | *p* (cluster-corrected) | Extent | *p* (voxel-corrected) | t(97) | X | Y | Z | region |
| --- | --- | --- | --- | --- | --- | --- | --- | --- |
| 3-back > 1-back | <0.001 | 41 | <0.001 | 10.51 | 45 | 41 | 23 | R DLPFC |
|  | <0.001 | 91 | <0.001 | 9.72 | -45 | 11 | 32 | L DLPFC |
|  | <0.001 | 94 | <0.001 | 9.57 | 42 | 32 | 35 | R DLPFC |
|  | <0.001 | 23 | <0.001 | 9.41 | 3 | 29 | 38 | R DLPFC |
|  | 0.017 | 2 | <0.001 | 9.15 | 42 | 50 | 20 | R DLPFC |
|  | <0.001 | 8 | <0.001 | 8.47 | -6 | 29 | 38 | L DLPFC |
|  | <0.001 | 29 | <0.001 | 7.72 | -45 | 29 | 23 | L DLPFC |
|  | 0.006 | 6 | <0.001 | 7.54 | 33 | 41 | 29 | R DLPFC |
|  | 0.026 | 1 | <0.001 | 6.68 | -42 | 50 | 17 | L DLPFC |
|  | 0.007 | 5 | 0.002 | 5.72 | 51 | 23 | 26 | R DLPFC |
|  | 0.026 | 1 | 0.014 | 5.18 | -9 | 35 | 29 | L DLPFC |

**Supplemental Table 3. DLPFC activation: n-back task.** Small volume (SV)-corrected activation for the contrast of interest in the n-back task, using a separate anatomical mask for left (L) and right (R) dorsolateral prefrontal cortex (DLPFC, here including Brodmann area 9 and Brodmann area 46) for the 3-back>1-back contrast (cluster-forming threshold *p*<0.05, FWE corrected at the voxel-level). DLPFC=dorsolateral prefrontal cortex.

1. Activation across groups: faces task

In the emotion processing task, the effect of fearful (versus neutral) faces (whole-brain p<0.05, voxel-level FWE-corrected) evoked bilateral activation in the fusiform gyri, as well as a large cluster in the lateral temporal cortex, and a cluster in the ventrolateral prefrontal cortex. There were no whole-brain significant results for the inverse contrast, or for the effect of happy (versus neutral) faces (or its inverse) at this threshold. The effect of faces in general (versus the fixation cross baseline) evoked widespread activation, including large clusters in the visual associative area, hippocampus, supplementary motor area, and orbitofrontal regions. The inverse contrast also evoked distributed activation, with the largest clusters in the sgACC and posterior cingulate, and smaller ones in sensory, parietal, and temporal regions. See Supplemental Table 4 for all whole-brain results.

We explored whether significant activation was present in our a priori ROIs using a small-volume (SV) correction for anatomical masks of the amygdalae and sgACC (cluster-forming threshold p<0.001, uncorrected; see Supplemental Table 5) for each contrast and its inverse. As expected from our whole-brain results, the effect of all faces vs fixation evoked significant activation in the bilateral amygdalae, and its inverse (fixation>faces) evoked significant activation in the sgACC. The fearful>neutral contrast also yielded significant activation in the bilateral amygdalae. The inverse emotion-specific contrasts (and the happy>neutral contrast) did not yield significant activation in any of our ROIs at this threshold.

See Supplemental Table 6 for co-primary outcome measures (mean, standard deviation, effect sizes, and one-sample t-statistics) for each group separately.

| Contrast | p (cluster level) | Extent (k) | p (voxel level) | t(98) | X | Y | Z | region |
| --- | --- | --- | --- | --- | --- | --- | --- | --- |
| fearful>neutral | <0.001 | 233 | <0.001 | 7.56 | 51 | -37 | 5 | R lateral temporal |
|  | <0.001 | 39 | <0.001 | 6.84 | -42 | -52 | -16 | L fusiform |
|  | <0.001 | 26 | <0.001 | 6.26 | 45 | -43 | -16 | R fusiform |
|  | <0.001 | 22 | <0.001 | 5.88 | 54 | 32 | 2 | R VLPFC |
|  | <0.001 | 45 | 0.001 | 5.83 | -51 | -49 | 5 | L lateral temporal |
|  | <0.001 | 12 | 0.004 | 5.49 | 51 | 14 | -19 | R temporal pole |
|  | 0.010 | 4 | 0.020 | 5.06 | 30 | -94 | 5 | R vis. assoc. |
| neutral>fearful | n/a | n/a | n/a | n/a | n/a | n/a | n/a | n/a |
| happy>neutral | n/a | n/a | n/a | n/a | n/a | n/a | n/a | n/a |
| neutral>happy | n/a | n/a | n/a | n/a | n/a | n/a | n/a | n/a |
| faces>fixation | <0.001 | 5558 | <0.001 | 26.85 | 36 | -85 | -7 | R vis. assoc. |
|  | <0.001 | 2571 | <0.001 | 15.17 | -24 | -31 | -1 | L hipp. |
|  | <0.001 | 491 | <0.001 | 9.36 | -39 | -1 | 17 | L premotor cortex |
|  | <0.001 | 104 | <0.001 | 8.74 | -6 | 8 | 50 | L SMA |
|  | <0.001 | 138 | <0.001 | 7.77 | -39 | -28 | 41 | L parietal |
|  | <0.001 | 34 | <0.001 | 7.7 | 3 | 44 | -22 | R OFC |
|  | <0.001 | 65 | <0.001 | 6.89 | 30 | 35 | -19 | R VLPFC |
|  | <0.001 | 18 | <0.001 | 5.52 | 0 | 14 | 11 | Bilateral caudate |
|  | 0.010 | 4 | <0.001 | 5.48 | -39 | 47 | 29 | L rostral PFC |
| fixation>faces | <0.001 | 2493 | <0.001 | 14.66 | 9 | 44 | -4 | sgACC |
|  | <0.001 | 1685 | <0.001 | 14.4 | -12 | -64 | 20 | L post.cingulate |
|  | <0.001 | 270 | <0.001 | 11.13 | 27 | 29 | 38 | R mPFC |
|  | <0.001 | 654 | <0.001 | 11.09 | 39 | -19 | 17 | R prim. sensory |
|  | <0.001 | 414 | <0.001 | 8.27 | -54 | -7 | -13 | L sup. temporal |
|  | <0.001 | 73 | <0.001 | 8.13 | 42 | -16 | 41 | R M1 |
|  | <0.001 | 49 | <0.001 | 7.01 | -57 | -58 | 26 | L parietal |
|  | <0.001 | 33 | <0.001 | 6.66 | 60 | -58 | 26 | R inferior parietal |
|  | <0.001 | 12 | <0.001 | 6.38 | -60 | -55 | -7 | L fusiform |
|  | 0.001 | 4 | 0.020 | 5.06 | 18 | 50 | 23 | R rostral PFC |

**Supplemental Table 4. Whole brain activation results: emotion processing task.** Whole brain activation (cluster-forming threshold *p*<0.05, FWE-corrected). R=right; L=left. VLPFC=ventrolateral prefrontal cortex; OFC=orbitofrontal cortex; mPFC=medial prefrontal cortex; SMA=supplementary motor area; sgACC=subgenual anterior cingulate cortex; vis. assoc.=visual associative; prim.=primary; sup.=superior; M1=primary motor cortex. Both cluster-level and voxel level *p*-values are whole-brain FWE corrected.

| Contrast | *p* (cluster-corrected) | Extent | p (voxel-corrected) | t(98) | X | Y | Z | region |
| --- | --- | --- | --- | --- | --- | --- | --- | --- |
| happy>neutral | 0.989 | 1 | 0.849 | 3.66 | 3 | 41 | 2 | sgACC |
|  | 0.989 | 1 | 0.969 | 3.43 | 0 | 14 | -7 | sgACC |
|  | 0.989 | 1 | 0.996 | 3.22 | -3 | 29 | 4 | sgACC |
| neutral>happy | n/a | n/a | n/a | n/a | n/a | n/a | n/a | n/a |
| fearful>neutral | 0.002 | 28 | <0.001 | 4.71 | -30 | -1 | -19 | L Amyg |
|  | 0.004 | 16 | 0.001 | 4.31 | 30 | 2 | -22 | R Amyg |
| neutral>fearful | n/a | n/a | n/a | n/a | n/a | n/a | n/a | n/a |
| faces>fixation | 0.001 | 38 | <0.001 | 9.98 | -24 | -7 | -13 | L Amyg |
|  | <0.001 | 51 | <0.001 | 9.24 | 27 | -4 | -19 | R Amyg |
| fixation>faces | <0.001 | 299 | <0.001 | 13.82 | 9 | 38 | -7 | sgACC |

**Supplemental Table 5. Amygdala and sgACC activation: faces task.** SV-corrected activation for all contrasts in the faces task using separate anatomical masks for the subgenual anterior cingulate cortex (sgACC), and right (R) and left (L) amygdalae (Amyg) (cluster-forming threshold *p*<0.001, uncorrected).

|  | *DLPFC ROI* | *Amygdala ROI* | *sgACC ROI* |
| --- | --- | --- | --- |
| *Controls* | M=0.316 (SD=0.302)  t(29)=5.71, p<0.001  Cohen’s d=1.06 | M=0.103 (SD=0.261)  t(29)=2.16, p=0.04  Cohen’s d=0.401 | M=-0.007 (SD=0.241)  t(29)=0.165, p=0.870  Cohen’s d=0.31 |
| *Relatives* | M=0.322 (SD=0.197)  t(28)=8.82, p<0.001  Cohen’s d=1.67 | M=0.114 (SD=0.388)  t(29)=1.61, p=0.118  Cohen’s d=0.299 | M=0.001 (SD=0.254)  t(29)=0.023, p=0.982  Cohen’s d=0.004 |
| *Patients* | M=0.153 (SD=0.279)  t(38)=3.43, p=0.001  Cohen’s d=0.556 | M=0.140 (SD=0.210)  t(38)=4.18, p<0.001  Cohen’s d=0.678 | M=-0.030 (SD=0.255)  t(38)=0.769, p=0.447  Cohen’s d=0.136 |

**Supplemental Table 6. Co-primary outcome measures (all tasks).** Means (M), standard deviations (SD), one-sample t-statistics, and effect sizes (Cohen’s d) for each co-primary outcome in each group: activation in the bilateral dorsolateral prefrontal cortex (DLPFC) during 3-back>1-back contrast; bilateral amygdala activation during fearful>neutral contrast; and sgACC activation during fearful>neutral). ROI=region of interest.

1. Whole-brain group differences: n-back task

In each task, for each contrast, we also ran an exploratory whole-brain ANOVAs (*F*-test) in SPM to test for group differences.

In the n-back task, no group differences survived whole-brain FWE correction (either cluster- or voxel-level). For completeness, we report all results exceeding a threshold of *p*<0.001 (uncorrected) in Table 7. Employing a SV correction using a bilateral DLPFC anatomical mask, the L DLPFC cluster did not survive FWE correction (either cluster- or voxel-level); for completeness, all SV-corrected results exceeding a threshold of p<0.001 (uncorrected) are reported in Supplemental Table 8.

| Contrast | p (cluster-corrected) | Extent | p (voxel-corrected) | F(2,95) | X | Y | Z | region |
| --- | --- | --- | --- | --- | --- | --- | --- | --- |
| 3-back>  1-back | 0.978 | 3 | 0.981 | 8.55 | -12 | -13 | 47 | Mid-cingulate |
|  | 0.990 | 2 | 0.982 | 8.54 | 15 | -10 | 35 | dACC |
|  | 0.990 | 2 | 0.990 | 8.32 | -21 | 32 | -7 | L VLPFC |
|  | 0.848 | 8 | 0.995 | 8.12 | -45 | 20 | 29 | L DLPFC |
|  | 0.978 | 3 | 0.997 | 7.99 | 36 | 23 | -7 | R insula |
|  | 0.997 | 1 | 0.998 | 7.84 | 36 | -37 | -10 | R hippocampus |
|  | 0.990 | 2 | 0.999 | 7.72 | -15 | -85 | -22 | L cerebellum |
|  | 0.997 | 1 | 0.999 | 7.67 | -42 | -1 | 32 | L premotor |
|  | 0.990 | 2 | 0.999 | 7.64 | -27 | -34 | 8 | L thalamus |
|  | 0.997 | 1 | 0.999 | 7.63 | 27 | -40 | -1 | R hippocampus |
|  | 0.997 | 1 | 0.999 | 7.58 | -39 | 17 | 14 | L VLPFC |

**Supplemental Table 7.** **Whole brain group differences: n-back task.** Whole brain group differences on the n-back task (*p*<0.001 uncorrected). Note: the F-test tests for effects in both directions (i.e., the contrast and its inverse). L=left; DLPFC=dorsolateral prefrontal cortex; dACC=dorsal anterior cingulate cortex; VLPFC=ventrolateral prefrontal cortex.

| Contrast | p (cluster-corrected) | Extent | p (voxel-corrected) | F(2,95) | X | Y | Z | region |
| --- | --- | --- | --- | --- | --- | --- | --- | --- |
| 3-back>  1-back | 0.530 | 1 | 0.624 | 7.59 | -45 | 17 | 26 | L DLPFC |

**Supplemental Table 8.** **SV-corrected group differences: n-back task.** SV-corrected group differences on the n-back task (*p*<0.001 uncorrected). Note: the F-test tests for effects in both directions (i.e., the contrast and its inverse). L=left; DLPFC=dorsolateral prefrontal cortex.

1. Whole-brain group differences: faces task

There were no whole-brain significant differences in the emotion processing task in the three ROIs (amygdalae and sgACC), corrected for 3 ROIs. For completeness we report all results exceeding a threshold of p<0.001 (uncorrected) in in Table 9. Employing a SV correction using anatomical masks of each ROI (amygdalae and sgACC), there were no clusters that survived, either cluster- or voxel-correction.

| Contrast | *p* (cluster-corrected) | Extent | *p* (voxel-corrected) | F(2,96) | X | Y | Z | region |
| --- | --- | --- | --- | --- | --- | --- | --- | --- |
| faces>baseline | 0.201 | 26 | 0.695 | 10.32 | -42 | -64 | -31 | L cerebellum |
|  | 0.995 | 1 | 0.996 | 8.03 | 21 | -22 | 38 | WM |
|  | 0.995 | 1 | 0.999 | 7.72 | -33 | -52 | 17 | L parietal |
| fearful>neutral | 0.951 | 4 | 0.959 | 12.83 | -27 | -16 | 20 | L dorsal thalamus |
|  | 0.986 | 2 | 0.188 | 8.76 | -3 | -34 | -43 | L pons |
| happy>neutral | 0.969 | 3 | 0.733 | 10.11 | -36 | -25 | 26 | L prim. sensory |
|  | 0.997 | 1 | 0.999 | 8.65 | 15 | -34 | 11 | R dorsal thalamus |
|  | 0.990 | 2 | 0.980 | 7.74 | 6 | -22 | -16 | Midbrain |

**Supplemental Table 9. Whole brain group differences: emotion processing task**. Whole brain group differences on the emotion processing task (*p*<0.001 uncorrected). Note: the *F*-test tests for effects in both directions (i.e., the contrast and its inverse). R=right; L=left; prim.=primary; WM=white matter.
